# Supplementary material for: Characterisation of an Isogenic Model of Cisplatin Resistance in Oesophageal Adenocarcinoma Cells
Source: Pharmaceuticals (Basel). 2019 Feb 20;12(1):33. doi: 10.3390/ph12010033 (PMC6469161; doi:10.3390/ph12010033)
Supplement: Supplementary file 1 [file pharmaceuticals-12-00033-s001.pdf]

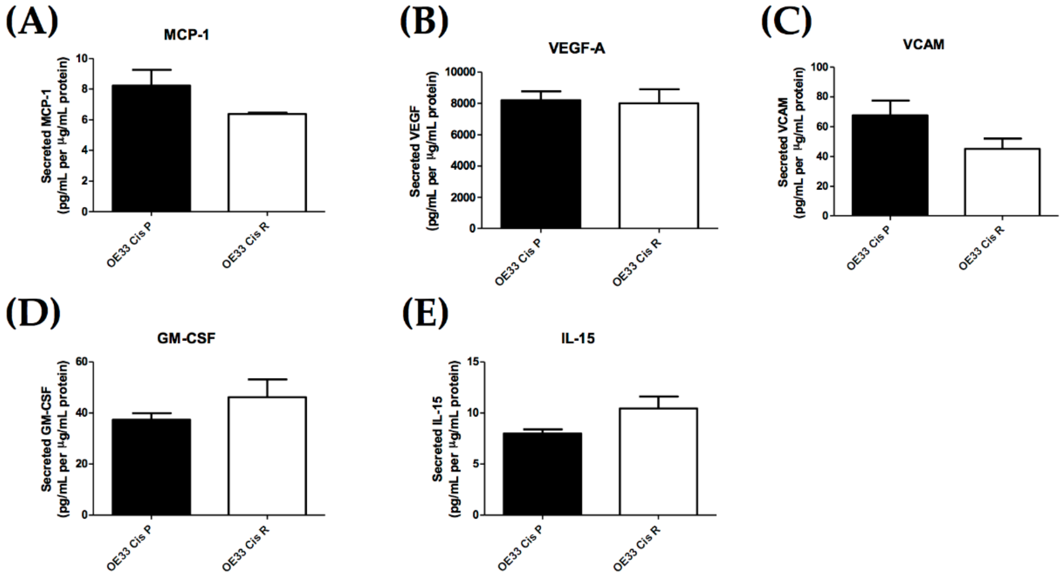

Supplemental Figure 1. Inflammatory protein in cisplatin sensitive (OE33 Cis P) versus cisplatin resistant (OE33 Cis R) OAC cells. The secreted levels of 47 proteins in Cis P and Cis R cells was evaluated by multiplex ELISA, 23 proteins were detected in supernatant of Cis P and Cis R cells, there was no significant difference in secreted levels of **(A)** Monocyte chemoattractant protein-1 (MCP-1) **(B)** Vascular endothelial growth factor (VEGF-A) **(C)** Vascular adhesion molecule 1 (VCAM-1) **(D)** Granulocyte-macrophage colony-stimulating factor (GM-CSF) **(E)** Interleukin 15 (IL-15) in OE33 Cis P and OE33 Cis R cells, all secretions normalised to protein content. (n = 4). Unpaired t-test. Date expressed as ± SEM.
